# Volatile aroma compounds of passion fruit seed Oils: HS-GC-IMS analysis and interpretation

**DOI:** 10.1016/j.fochx.2024.101212

**Published:** 2024-02-09

**Authors:** Lili Zheng, Shenwan Wang, Yang Yang, Xiaoyan Zheng, Dao Xiao, Binling Ai, Zhanwu Sheng

**Affiliations:** a Institute of Tropical Bioscience and Biotechnology, Chinese Academy of Tropical Agricultural Sciences, Haikou, Hainan 571101, China; b Agricultural Products Processing Research Institute, Chinese Academy of Tropical Agricultural Sciences, Zhanjiang, Guangdong 524000, China; c Huazhong Agricultural University, College of Food Science and Technology, Wuhan, Hubei 430070, China

**Keywords:** Passion fruit seed oil, Cold-pressed, Volatile compounds, HS-GC-IMS, Aroma

## Abstract

The physicochemical properties, fatty acid composition and volatile aroma compounds of cold-pressed passion fruit seed oils were analyzed. The oils were rich in linoleic acid, oleic acid and volatile compounds. A total of 108 volatile compounds including 17 aldehydes, 23 alcohols, 21 esters, 19 ketones, 6 acids, 9 alkenes, 5 pyrazines and 8 others were identified using HS-GC-IMS. The significant differences of volatile compounds in the purple and yellow passion fruit seed oils were observed via the GalleryPlot graph and distinguished by principal component analysis. The results showed that acids, alcohols, esters and ketones were major aromatic compounds in purple passion fruit seed oils, which contribute to flavors such as flowery, fruity, creamy, yogurt. Whereas the contents of aldehydes, pyrazines, alkenes were higher in yellow passion fruit seed oils, which contributes to fatty and nutty odors. The findings filled in our understanding of volatilization characteristics in passion fruit seed oils.

## Highlights

• Passion fruit seed oil by cold-pressed is rich in polyunsaturated fatty acids.
• A total of 108 volatile aroma compounds were identified.
• Acids, alcohols, esters and ketones were higher in purple passion fruit seed oil.
• Aldehydes, pyrazines, alkenes were higher in yellow passion fruit seed oil.
• Rich of aroma components are rare and surprising in cold-pressed oil.

## Graphical abstract

**Figure:**
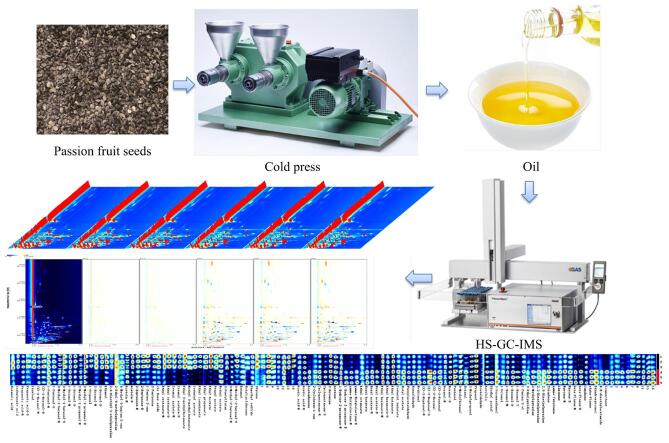

## 1 Introduction

Passion fruits are rich in nutritional value, flavor, and bioactive constituents such as phenolic compounds, carotenoids, vitamins, and dietary fibers. They are usually consumed fresh; however, in the last decades they have found applications in food processing, pharmaceutical, and cosmetic industries (Corrêa et al., 2016). Based on their intense, delightful, and peculiar aroma, passion fruits are suitable for fruit juices, jams, desserts, and fermented foods and are widely popular among consumers worldwide. In recent years, the annual international demand for passion fruit juice increased by 15 %–20 % (Zhao et al., 2023). Even though peels and seeds are important by-products of passion fruit processing, they are often discarded by processing enterprises. The pulp corresponds to 23 % of the fruit’s weight, whereas the peel and the seed contribute to 50 % and 27 % of its mass, respectively (Silva, Rodrigues, & Bottoli, 2021). The peels can be used as fertilizer and fodder (Sena et al., 2015). Several studies have reported the nutritional and functional values of edible pectin/polysaccharides and phenolic compounds extracted from passion fruit peels, which can be used as consistency agents and antioxidant components (Liang et al., 2022, da Costa et al., 2023).While passion fruit, in fact, contains 14.9 %–30.1 % oil(Fonseca, Geraldi, Junior, Silvestre, & Rocha, 2022). Depending on the diversity of species, regions, and climates, its seed oil is rich in unsaturated fatty acids, including linoleic acid (53.0–75.1 % (w/w) of total FA content), oleic acid (13.6–31.2 % (w/w) of total FA content), and smaller amounts of linolenic, palmitoleic, and gadoleic acids (Fonseca et al., 2022). The unsaturated fatty acids in the seed oil are important substances for the human health and can promote the growth and development of infant brains, enhance the permeability of cells, reduce the accumulation of cholesterol, and prevent myocardial vascular blockage and arteriosclerosis (Pereira, Cruz, Corrêa, Sanches, Campelo, & Bezerra, 2023). Based on these functional properties and beneficial efficacy, passion fruit seed oil can be used as a high-quality functional healthy edible oil, exhibiting promising potential worldwide.

Extraction methods for passion fruit seed oil include pressing, Soxhlet extraction, steam distillation extraction, ultrasonic-assisted extraction, and supercritical carbon dioxide extraction (dos Santos et al., 2019, Le et al., 2023, Wongwaiwech et al., 2019). Solvent extraction or mechanical pressing are commonly used techniques in production; however, the residual solvent and low extraction rate are shortcomings that need to be addressed (Ribeiro et al., 2020). Low-temperature cold pressing technology, which is an advanced extraction method for healthy edible oils, limits the activity of enzymes and reduces the transfer of unwanted compounds from seed to oil (Rombaut et al., 2015, Chew, 2020). The material is physically pressed under the non-heating treatment conditions without heating to reduce the loss of fatty acids and bioactive compounds. The nutrients and functional components are therefore maintained in cold-pressed oil (Chew, 2020, Zeng et al., 2022, Xu et al., 2022).

An interesting discovery was found that is a super attractive aroma filled the air in our practice of cold-pressed passion fruit seed oil. This aroma which is not observed in extractions of other oils (peanut, camellia, and sesame oils) using the same process. Cold-pressing equipment involves pressing the fruit seeds by screw extrusion and no heat treatment is performed before the seeds enter the machine. Heating is only applied in the material residue continuous discharge port (120–140 °C) for extrusion forming. Therefore, the temperature of cold-pressed oil does not exceed 60 °C, which is not conducive to the release of volatile aromas in the oil (Xu, Wang, et al., 2023). Aroma compounds are usually produced via the Maillard reaction, lipid oxidation, Strecker degradation of amino acids, and sugar degradation (Mao et al., 2019, Zhang et al., 2022, Zhang et al., 2022). Lower temperatures are not conducive to these reactions, which is also the main reason for the lack of aroma in cold-pressed oils. However, passion fruit seed oil is a special case since volatile components contribute to the intense aroma of passion fruit seed oil at low temperatures. The oil aroma characteristics and differences between the different varieties of passion fruit seed oil remain unclear.

Herein, purple and yellow passion fruits (*P. edulis f. edulis* and *P. edulis f. flavicarpa*, respectively) were used as raw materials to obtain two cold-pressed oils by an automatic cold press machine. Headspace gas chromatography-ion migration spectrometry (HS-GC-IMS) was used to analyze the volatile metabolites and create aroma fingerprints of the two passion fruit seed oils. HS-GC-IMS combines the advantages of high separation via gas chromatography and high sensitivity via ion migration spectrometry to rapidly separate and quantify trace volatile organic compounds without any special and additional preparation. This chromatographic separation is a powerful method for resolving complex mixtures, enabling precise compound identification, including isomer identification, which acts as an effective complement to volatile aroma profiling in GC–MS. Furthermore, we explored the characteristic aromatic substances using dynamic principal component analysis (PCA). The fatty acid composition and physicochemical parameters of the two cold-pressed passion fruit seed oils were analyzed to determine their contribution to the volatile aroma substances. This study provides a theoretical basis for the deep processing industry of passion fruit seed oils and promotes the utilization of passion fruit processing by-products to enhance its value chain.

## 2 Materials and methods

### 2.1 Materials

Passion fruit seeds were obtained from the waste of a frozen juice factory (Hainan Bingguo Division Biotechnology Co., LTD) in Changjiang county, Hainan province, China. The BF_3_·MeOH solution (14 %) was purchased from Sigma–Aldrich (St. Louis, MO, USA). All the other chemicals were of analytical grade or higher.

### 2.2 Preparation of passion fruit seed oil

Passion fruit seeds were washed, dried, and pressed using an automatic cold-pressing machine (Germany, KOMET, DD85G). The slag exit diameter was 15 mm, equipped with a heating jacket at the outer surface of the waste slag exit with setting the preheating temperature to 130 ± 5 °C for 30 min, then start to fill seeds and press oil. The temperature was maintained steady until the end of the pressing process to ensure smooth extrusion of the waste residue. The oil temperature was not directly affected by the heating temperature, which was kept below 60 °C during screw extrusion. The cold pressed passion fruit seed oil was filtered to remove impurities, transferred into amber glass bottles, and stored at 4 °C.

### 2.3 Physical and chemical properties

The acid value, peroxide value, crude protein content, and iodine value were determined by following the AOAC official methods 969.17, 965.33, 2001.11, and 920.160, respectively (Aoac, 2005). A saponification test was performed in accordance with the National Standards of the People's Republic of China (GB/T 5534-2008).

### 2.4 Fatty acids content

The fatty acid composition of passion fruit seed oil was determined by transmethylation of fatty acids to produce the corresponding fatty acid methyl esters (Salas et al., 2023) with some modifications. The oil sample (5–10 mL) was mixed with 2 mL of 14 % BF_3_·MeOH, followed by methylation at 60 °C for 30 min to convert all fatty acids to methyl esters (Ackman, 1998). The resulting fatty acid methyl esters were extracted with 2 mL of *n*-hexane and then analyzed using GC–MS (Agilent 7890–5975 Series GC System (Santa Clara, CA, USA)), equipped with a HP-5MS (60 m × 0.25 mm, 0.25 μm) column. Helium was used as the carrier gas at a flow rate of 1.0 mL/min. The injection temperature was set to 280 ℃, and the column temperature followed a ramping procedure. First, maintain the initial temperature of 120 °C for 1 min; increase the temperature to 170 ℃ for 5 min at a rate of 6 °C/min; increase the temperature to 215 ℃ for 12 min at a rate of 2.5 ℃/min; increase the temperature to 230 °C for 10 min at a rate of 4 °C/min; finally, increase the temperature to 280 °C for 15 min at a rate of 10 ℃/min. The injection volume was 1 μL, the split ratio was 20:1, and the MS conditions were as follows. Ion source temperature: 200 °C; quadrupole temperature: 150 °C; connecting line temperature: 260 °C; electron bombardment energy: 70 eV; quality scanning range: 40–550 *m*/*z*; solvent removal time: 4.4 min. Fatty acid esters were identified using the NIST 11 spectrum database retrieval and were compared with 37 fatty acid methyl ester standard spectra and retention times. Methyl undecanoate was used as an internal standard and was quantitatively analyzed under the test conditions. These results are reported as weight percentages. The relative concentrations (%) of fatty acids were quantified using peak area normalization.

### 2.5 HS-GC-IMS analysis

The volatile compounds in two passion fruit seed oil samples were detected using a GC-IMS instrument (Flavourspec®, G.A.S, Dortmund, Germany). Briefly, 2 g of the oil sample was placed in a 20-mL headspace vial and incubated at 80 °C for 20 min with an agitation speed of 500 rpm. Then, the headspace sample (0.5 mL) was added into the injection port using a heated syringe at 85 °C. The separation of the compounds was achieved using a chromatographic column (WAX, 15 m × 53 mm × 1 μm, RESTEK Company) at a temperature of 60 °C, whereas the ion mobility spectrum temperature was set at 45 °C. High-purity nitrogen was employed as the carrier gas at the following gradient: 2 mL/min for 2 min, increased to 10 mL/min within 10 min, increased to 100 mL/min within 20 min, and held at 100 mL/min for 20 min. The drift-gas flow rate was maintained at 150 mL/min.

The retention index (RI), retention time (Rt), and migration time (Dt) were combined to qualitatively characterize the volatile compounds in the passion fruit seed oil samples. The volatile compounds were differentiated using the analysis software of the instrument (observation of analytical spectra and data, application software built-in NIST library, and IMS database for qualitative analysis of substances) and three plug-ins (Reporter, Gallery Plot, Dynamic PCA). The fingerprints and differential spectrograms were analyzed using Gallery Plot and Reporter plug-ins, respectively.

### 2.6 Statistical analysis

The physical and chemical properties and fatty acid composition of all oil samples were tested thrice. Pie charts, histograms, and box plots were plotted using the Origin software (OriginLab, USA). The GC-IMS data were analyzed using the plug-ins of the supporting software (G.A.S., Dortmund, Germany).

## 3 Results and discussion

### 3.1 Physical and chemical properties

The decomposition and oxidation of plant oils occur slowly upon contact with lipidases or O_2_ in the air, resulting in an unpleasant odor and taste (Naebi, Torbati, Azadmard-Damirchi, Siabi, & Savage, 2022). Acid values can be used to assess the rancidity due to enzymatic or chemical oxidation. The physical and chemical properties of the two passion fruit oils are presented in Table 1; the cold-pressed oils exhibited an acid value of approximately 1 mg KOH/g, which is far below the limits of the Codex standard (4 mg KOH/g for virgin and cold-pressed oils) (Alimentarius, 1999). Such a finding was lower compared to the values reported by Le (2023) for the hydraulic oil pressing of purple passion fruit seed oil (2.039–2.6 mg KOH /g). The peroxide value of passion fruit seed oil was extremely low (0.02 g/100 g), indicating that the oil was stable and not prone to rancidity. The iodine value is a measure of the degree of unsaturation. The high iodine value of passion fruit seed oil (approximately 139.50 g/100 g) indicates its high unsaturation, containing more conjugated double bonds in the fatty acid main chain. In general, low acid and peroxide values combined with a high iodine value allow oils to withstand oxidative deterioration and lipolytic hydrolysis (Le et al., 2023, Alireza et al., 2010). The saponification values of the two passion fruit seed oils were lower than those of the most common plant oils, indirectly reflecting their molecular weights. The crude protein contents of the two oils were 62.00 and 57.50 mg/100 g, which were attributed to the extrusion process. The oxidation and Maillard reactions of proteins may also affect the sample’s odor.

**Table 1:** Physical and chemical properties of two passion fruit seed oils.

Physical and chemical index	Purple	Yellow
Acid value (mg KOH /g)	1.11	0.97
Peroxide value (mg/100 g)	17.69	26.97
Crude protein content (mg/100 g)	62.00	57.50
Iodine value (g/100 g)	139.53	139.45
Saponification value (mg KOH/g)	74.93	73.06

### 3.2 Fatty acid composition

As presented in Table 2, the fatty acid composition of the two cold-pressed passion fruit seed oils was similar, however, differences were also observed. The purple and yellow passion fruit seed oils contained 85.97 % and 83.55 % of unsaturated fatty acids, respectively; 70.36 % and 68.36 % linoleic acid (polyunsaturated fatty acids) and 13.40 % and 13.50 % oleate acid (monounsaturated fatty acids), respectively. The linoleic acid content of passion fruit seed oil was higher than that of grape seed oil (66.85 %), walnut oil (57.30 %) (Xue et al., 2023), and cold-pressed sesame oil (42.09–43.38 %) (Huang et al., 2023). Furthermore, the linoleic acid content of common vegetable oils, such as soybean oil, peanut oil, and rapeseed oil is less than one half of the total fatty acid content (Zhang et al., 2014). Linoleic acid is beneficial to human health, able to reduce or eliminate cancer, prevent heart disease, improve immune function, and alter the body composition to treat obesity or build a lean body mass (Whigham, Cook, & Atkinson, 2000). The health benefits of the omega-6 fatty acid family include linoleic acid and its long-chain derivatives (Chew, 2020). Cis-11-eicosenoate is a specific unsaturated fatty acid, which is present in low amounts in purple passion fruit seed oil (0.14 %) and absent in yellow passion fruit seed oil. In contrast, apart from saturated fatty acids such as palmitate, stearate, behenate, and tetracosanoic acid, yellow passion fruit seed oil also contains 0.13 % myristic acid and 0.13 % arachidonic acid. These two ingredients are often used in cosmetics to moisturize the skin; therefore, yellow passion fruit seed oil is promising for applications in the cosmetics industry.

**Table 2:** Fatty acid composition of the two passion fruit seed oils.

Fatty acid	Purple /%	Yellow /%
Myristate; C14:0	——	0.13
Palmitoleate; C16:1 (*cis*-9)	0.15	0.11
Palmitate; C16:0	11.32	12.87
Linoleate; C18:2	70.36	68.36
Oleate; C18:1(*cis*-9)	13.40	13.50
Elaidate; C18:1T(*trans*-9)	1.12	0.99
Stearate; C18:0	2.46	2.94
Cis-11,14-Eicosadienoic acid ester; C20:2(*cis*-11,14)	0.81	0.70
Cis-11-Eicosenoate; C20:1	0.14	——
Behenate; C22:0	0.10	0.11
LignocerateI; C24:0	0.15	0.16
Arachidate;C20:0	——	0.13
Unsaturated fatty acid	85.97	83.55
Saturated fatty acid	14.03	16.45

### 3.3 Volatile organic compounds analysis via HS-GC-IMS

Compared to single GC, the HS-GC-IMS technology effectively enhances the sensitivity of complex mixture analysis and has been extensively used to determine volatile flavor ingredient fingerprints for food classification and adulteration detection (Han et al., 2022).

**Fig. 1:**
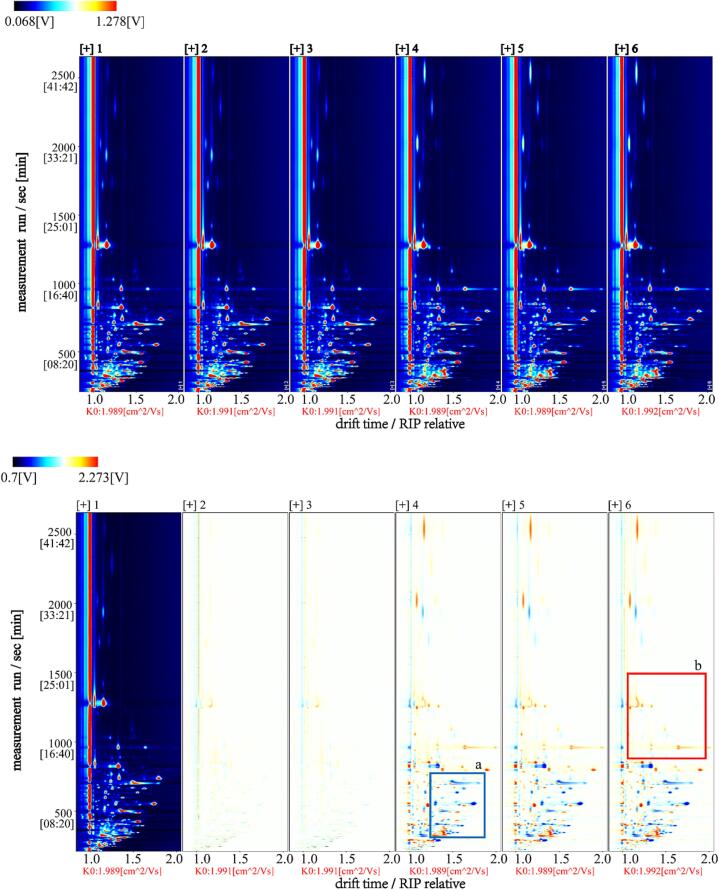
HS-GC-IMS spectrogram. A: top view. The red vertical line at horizontal coordinate 1.0 is the reactive ion peak (RIP) in the blue background. Each point in the graph represents a volatile organic compound. B: Difference view. With 1 as the reference, the signal peak in 1 is subtracted from the rest of the spectra and the difference in the spectra of the two samples was obtained. The blue area indicates that the substance is lower than 1(a), and the red area indicates that the substance is more than 1(b). The darker the color, the greater the difference. (For interpretation of the references to color in this figure legend, the reader is referred to the web version of this article.)

As shown in Fig. 1, these signals approximately covered the HS-GC-IMS region in which passion fruit seed oil volatile compounds. The Y-axis shows the retention time of the gas chromatograph and the x-axis represents the ion migration time. The distributions of volatile compounds in the seed oils of the two passion fruit types were similar (Fig. 1A). Each point on the graph represents a volatile organic compound. The degree of color represents the concentration of the substance: white indicates a lower concentration, red indicates a higher concentration, and the darker color indicates a greater concentration. Fig. 1A shows dense signals indicating the abundance of volatile compounds in passion fruit seed oil. Most signals appeared between 0 and 1000 s with a drift time of 1.0 and 1.75 s. The contents of volatile compounds in the two types of passion fruit seed oils exhibited obvious differences, as shown in zones A and B (Fig. 1B). However, it is difficult to accurately determine the types of flavor substances in the present stage.

### 3.4 Fingerprints of volatile compounds

**Fig. 2:**
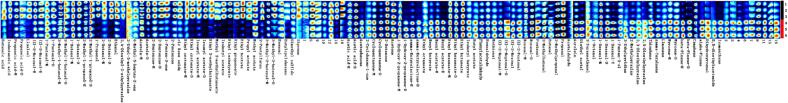
GalleryPlot graph of the two oils (1, 2, 3: purple passion fruit seed oil and 4, 5, 6: yellow passion fruit seed oil). Each row represents all the signal peaks selected in a sample, and each column represents the signal peak of the same volatile compounds in a different sample. The letters M and D in the parentheses represent the compound's monomer and dimer. The degree of color reflects the intensity of the signal. Increase in the contents of volatile compounds is indicated with a color change from blue to red. (For interpretation of the references to color in this figure legend, the reader is referred to the web version of this article.)

The depth of color in Fig. 2, reflects the intensity of the signal. A total of 108 volatile compounds were identified in the passion fruit seed oil samples. The contents of acids, alcohols, esters, and ketones were higher in the purple passion fruit seed oil samples (A: 1, 2, 3), whereas the contents of aldehydes, pyrazines, and alkenes were higher in the yellow passion fruit seed oil samples (B: 4, 5, 6). The volatile compounds detected in the two seed oil types were significantly different. The purple passion fruit seed oil is rich in butyric acid, isobutyric acid, propanoic acid, linalool, Z-3-hexenol, 1-amyl alcohol, 3-methyl-1-butanol, 1-butanol, 2-methyl-1-propyl alcohol, 1-propyl alcohol, 2-butanol, and ethyl alcohol. In addition, it contains more ketones, including 6-methyl-5-heptene-2-ketone, acetoin, 2-heptanone, 1-pentene-3-ketone, 2-pentanone, and esters, including ethyl crotonate, isoamyl acetate, ethyl 3-methylbutyrate, methyl 3-methylbutyrate, isobutyl acetate, ethyl isobutyrate, ethyl propionate, ethyl acetate, propyl acetate, and methyl acetate. Most ketones are oxidation products of oleic and linoleic fatty acids, which exude fragrances such as flowery, fruity, creamy, and yogurt (Xu, Wang, et al., 2023). Purple passion fruit seed oil exhibited high contents of 2,6-dimethyl-3-ethylpyrazine, 2-ethyl-5-methylpyrazine, *cis*-rose ether, dimethyl sulfide, 3-methyl-2-butenal, aldehyde, butylcyclohexane, and 2-amylfuran. Aldehydes are mainly involved in the fatty smell, whereas a nutty odor originates from pyrazines. Furthermore, furans mainly produce caramel-like fragrances (Zhang et al., 2023).

By contrast, the levels of aldehydes in yellow passion fruit seed oil including phenylacetaldehyde, benzaldehyde, methyl thiopropionaldehyde, E-2-heptenal, E-2-hexenal, E-2-pentenal, hexal, pentylaldehyde, 3-methylbutyral, butyral, 2-methylpropanal, propanal, acetaldehyde, acrolein, acetal, and pyrazines including 2-methylpyrazines, 2, 3-dimethylpyrazines, 2, and 5-dimethylpyrazines were high. High levels were also observed for alkenes including gamma-terpinene, limonene, laurene, β-pinene, camphene, and α-pinene. Because of their low odor threshold, aldehydes play an important role in the flavor of oil samples. Some aldehydes, such as pentanal and (E)-2-heptenal, are oxidation products of fatty acids that give a fatty odor (Chang et al., 2020, Xu et al., 2023). Pyrazines usually exhibit odors such as roasted, sauce, nutty, and bread (Zhang et al., 2023). The levels of acetic acid, 3-furanol, 1-octanol, 1-hexanol, 1-pentene-3-ol, γ-butyrolactone, ethyl caprylate, hexyl butyrate, hexyl acetate, ethyl caproate, ethyl butyrate, isobutyl butyrate, and butyl acetate were high. Furthermore the levels of ketones including acetophenone, 2-cyclohexene-1-one, cyclopentanone, 2-butanone, acetone, and 1-hydroxy-2-acetone were also high. Moreover, the contents of thiophene, γ-terpinene, limonene, laurene, β-pinene, camphene, α-pinene, dihydrolauryl alcohol, N, *N*-dimethylacetamide, and isomenthone were higher. The signature volatile odors mainly include heterocyclic aroma-active compounds, such as thiophene, which have a roasted garlic odor. Alkenes like γ-terpinene give a caramel, fruity, and citrusy smell (Yin, Ma, Li, Liu, & Shi, 2021), whereas β-pinene has a woody odor (Xu, Wang, et al., 2023). Limonene, which has a fruity aroma, is also a key flavor compound in vegetables (Yang et al., 2022).

Table 3 presents the retention index and migration time of all substances, the peak volume of the different substances in each sample, and similarity data between the samples. A total of 108 volatile aroma compounds were identified in the two types of oils using HS-GC-IMS: 19 ketones, 17 aldehydes, 23 alcohols, 21 esters, 6 acids, 9 alkenes, 5 pyrazines, and 8 others. Passion fruit seed oil is rich in aroma compounds, exhibiting a higher aroma content compared to that of common plant and vegetable oils (cold-pressed and hot-pressed): Olive oil contains 25 volatile compounds (del Mar Contreras, Arroyo-Manzanares, Arce, & Arce, 2019), green plum (*Buchanania obovata*) seed oil contains 42 volatile compounds (Xu, Wang, et al., 2023), commercia sesame oil contains 79 volatile compounds (Dou et al., 2022), cold-pressed walnut oil contains 83 volatile compounds (Xu, Bi, et al., 2023), camellia (Camellia oleifera Abel.) oil contains 80 volatile compounds (He, Wu, & Yu, 2021), cold-pressed peanut oil contains 92–95 volatile compounds, whereas hot-pressed peanut oil contains 108 (Hu et al., 2023).

**Table 3:** List of volatile compounds.

Count	Compound	Formula	MW	RI	Rt [*sec*]	Dt [RIPrel]	Comment
Ketones:19							
1	Acetophenone	C_8_H_8_O	120.2	1816.0	2523.343	1.17441	
2	γ-Butyrolactone	C_4_H_6_O_2_	86.1	1709.9	2004.324	1.09241	Monomer
3	γ-Butyrolactone	C_4_H_6_O_2_	86.1	1712.1	2013.853	1.30434	Dimer
4	2-Cyclohexen-1-one	C_6_H_8_O	96.1	1456.1	1155.43	1.10346	
5	6-Methyl-5-hepten-2-one	C_8_H1_4_O	126.2	1347.8	913.531	1.18104	
6	1-Hydroxy-2-propanone	C_3_H_6_O_2_	74.1	1316.3	853.186	1.06498	Monomer
7	1-Hydroxy-2-propanone	C_3_H_6_O_2_	74.1	1315.7	852.08	1.2301	Dimer
8	Cyclopentanone	C_5_H_8_O	84.1	1200.1	669.241	1.10888	Monomer
9	Cyclopentanone	C_5_H_8_O	84.1	1200.7	670.157	1.33591	Dimer
10	2-Heptanone	C_7_H_14_O	114.2	1193.5	660.191	1.26454	Monomer
11	2-Heptanone	C_7_H_14_O	114.2	1194.0	660.917	1.63505	Dimer
12	1-Penten-3-one	C_5_H_8_O	84.1	1031.6	395.488	1.29469	
13	2-Pentanone	C_5_H_10_O	86.1	1001.3	361.533	1.36776	
14	2-Butanone	C_4_H_8_O	72.1	919.5	299.552	1.24692	
15	Acetone	C_3_H_6_O	58.1	844.9	253.201	1.11624	
16	Isomenthone	C_10_H_18_O	154.3	1494.4	1255.697	1.3404	
17	Acetoin	C_4_H_8_O_2_	88.1	1299.9	823.322	1.06345	Monomer
18	Acetoin	C_4_H_8_O_2_	88.1	1299.9	823.322	1.32948	Dimer
19	Methional	C_4_H_8_OS	104.2	1475.4	1205.04	1.09953	

Aldehydes:17							
1	Phenylacetaldehyde	C_8_H_8_O	120.2	1767.5	2271.13	1.25951	
2	Benzaldehyde	C_7_H_6_O	106.1	1550.5	1418.363	1.15652	
3	(E)-2-Heptenal	C_7_H_12_O	112.2	1332.2	883.155	1.26068	Monomer
4	(E)-2-Heptenal	C_7_H_12_O	112.2	1332.2	883.049	1.67042	Dimer
5	(E)-2-Hexenal	C_6_H_10_O	98.1	1233.3	716.866	1.18283	
6	3-Methyl-2-butenal	C_5_H_8_O	84.1	1214.4	689.39	1.09684	Monomer
7	3-Methyl-2-butenal	C_5_H_8_O	84.1	1215.7	691.222	1.35999	Dimer
8	(E)-2-Pentenal	C_5_H_8_O	84.1	1151.4	575.252	1.11117	
9	Hexanal	C_6_H_12_O	100.2	1101.1	486.683	1.26454	Monomer
10	Hexanal	C_6_H_12_O	100.2	1102.4	488.861	1.56368	Dimer
11	Pentanal	C_5_H_10_O	86.1	1007.3	368.001	1.4338	
12	Butanal	C_4_H_8_O	72.1	890.6	280.688	1.28064	
13	Acrolein	C_3_H_4_O	56.1	870.6	268.292	1.06004	
14	Propanal	C_3_H_6_O	58.1	820.6	239.727	1.14434	
15	2-Methylpropanal	C_4_H_8_O	72.1	834.3	247.272	1.28205	
16	Acetaldehyde	C_2_H_4_O	44.1	769.9	213.856	0.97854	
17	Nonanal	C_9_H_18_O	142.2	1402.0	1027.506	1.47244	

Alcohols:23							
1	3-Furanmethanol	C_5_H_6_O_2_	98.1	1801.5	2445.03	1.1026	
2	Linalool	C_10_H_18_O	154.3	1643.4	1735.136	1.22487	
3	1-Octanol	C_8_H_18_O	130.2	1654.1	1775.633	1.47754	
4	(Z)-3-Hexenol	C_6_H_12_O	100.2	1402.8	1029.253	1.23911	Monomer
5	(Z)-3-Hexenol	C_6_H_12_O	100.2	1402.8	1029.253	1.51452	Dimer
6	1-Hexanol	C_6_H_14_O	102.2	1369.9	958.374	1.33202	Monomer
7	1-Hexanol	C_6_H_14_O	102.2	1370.6	959.82	1.64228	Dimer
8	1-Hexanol	C_6_H_14_O	102.2	1369.9	958.374	1.99401	Trimer
9	1-Pentanol	C_5_H_12_O	88.1	1266.6	768.154	1.25507	Monomer
10	1-Pentanol	C_5_H_12_O	88.1	1266.1	767.238	1.5079	Dimer
11	3-Methyl-1-butanol	C_5_H_12_O	88.1	1221.4	699.464	1.24647	Monomer
12	3-Methyl-1-butanol	C_5_H_12_O	88.1	1222.7	701.296	1.4907	Dimer
13	1-Penten-3-ol	C_5_H_10_O	86.1	1177.0	626.07	0.94414	
14	1-Butanol	C_4_H_10_O	74.1	1161.9	595.579	1.18406	Monomer
15	1-Butanol	C_4_H_10_O	74.1	1162.3	596.306	1.37994	Dimer
16	2-Methyl-1-propanol	C_4_H_10_O	74.1	1109.9	501.203	1.17191	Monomer
17	2-Methyl-1-propanol	C_4_H_10_O	74.1	1109.9	501.203	1.37083	Dimer
18	1-Propanol	C_3_H_8_O	60.1	1059.3	429.443	1.11343	
19	2-Butanol	C_4_H_10_O	74.1	1041.5	407.345	1.15137	Monomer
20	2-Butanol	C_4_H_10_O	74.1	1041.1	406.806	1.32139	Dimer
21	Ethanol	C_2_H_6_O	46.1	950.3	321.111	1.13451	
22	3-Methylbutanal	C_5_H_10_O	86.1	933.6	309.253	1.4057	
23	Dihydromyrcenol	C_10_H_20_O	156.3	1497.5	1264.267	1.22678	

Esters:21							
1	Ethyl octanoate	C_10_H_20_O_2_	172.3	1451.4	1143.854	1.48667	
2	Hexyl butyrate	C_10_H_20_O_2_	172.3	1426.8	1084.322	1.48863	
3	Hexyl acetate	C_8_H_16_O_2_	144.2	1284.3	796.776	1.39063	Monomer
4	Hexyl acetate	C_8_H_16_O_2_	144.2	1283.6	795.67	1.90128	Dimer
5	Ethyl hexanoate	C_8_H_16_O_2_	144.2	1246.7	737.015	1.34279	Monomer
6	Ethyl hexanoate	C_8_H_16_O_2_	144.2	1246.7	737.015	1.80201	Dimer
7	Ethyl crotonate	C_6_H_10_O_2_	114.1	1181.1	634.782	1.18558	Monomer
8	Ethyl crotonate	C_6_H_10_O_2_	114.1	1180.8	634.056	1.55305	Dimer
9	Isoamyl acetate	C_7_H_14_O_2_	130.2	1139.8	553.473	1.30554	Monomer
10	Isoamyl acetate	C_7_H_14_O_2_	130.2	1139.4	552.747	1.74894	Dimer
11	Ethyl 3-methylbutanoate	C_7_H_14_O_2_	130.2	1081.5	458.547	1.26659	
12	Ethyl butanoate	C_6_H_12_O_2_	116.2	1053.4	421.898	1.56027	
13	Methyl 3-methylbutanoate	C_6_H_12_O_2_	116.2	1036.6	401.417	1.53076	
14	Isobutyl acetate	C_6_H_12_O_2_	116.2	1030.2	393.871	1.61085	
15	Ethyl isobutyrate	C_6_H_12_O_2_	116.2	984.0	346.442	1.56027	
16	Ethyl propanoate	C_5_H_10_O_2_	102.1	976.4	340.513	1.45067	
17	Ethyl Acetate	C_4_H_8_O_2_	88.1	901.5	287.695	1.33544	
18	Methyl acetate	C_3_H_6_O_2_	74.1	857.0	260.207	1.19914	
19	Isobutyl butyrate	C_8_H_16_O_2_	144.2	1172.7	617.271	1.33259	
20	Butyl acetate	C_6_H_12_O_2_	116.2	1089.1	469.114	1.23785	
21	Propyl acetate	C_5_H_10_O_2_	102.1	995.6	355.621	1.47054	

Acids:6							
1	Isobutanoic acid	C_4_H_8_O_2_	88.1	1692.6	1930.476	1.15966	
2	Propanoic acid	C_3_H_6_O_2_	74.1	1636.4	1708.932	1.11279	Monomer
3	Propanoic acid	C_3_H_6_O_2_	74.1	1637.1	1711.314	1.26562	Dimer
4	Butanoic acid	C_4_H_8_O_2_	88.1	1731.3	2099.612	1.15966	
5	Acetic acid	C_2_H_4_O_2_	60.1	1501.9	1276.147	1.05826	Monomer
6	Acetic acid	C_2_H_4_O_2_	60.1	1503.0	1279.455	1.15259	Dimer
Alkenes:9							
1	γ-Terpinene	C_10_H_16_	136.2	1260.9	758.995	1.21551	
2	Myrcene	C_10_H_16_	136.2	1175.6	623.167	1.21746	Monomer
3	Myrcene	C_10_H_16_	136.2	1175.6	623.327	1.28462	Dimer
4	β-Pinene	C_10_H_16_	136.2	1133.8	542.583	1.21443	Monomer
5	β-Pinene	C_10_H_16_	136.2	1133.5	542.087	1.63052	Dimer
6	a-Pinene	C_10_H_16_	136.2	1029.3	392.793	1.23006	
7	Camphene	C_10_H_16_	136.2	1066.0	438.043	1.19924	
8	Limonene	C_10_H_16_	136.2	1205.4	676.606	1.21838	
9	Styrene	C_8_H_8_	104.2	1269.2	772.197	1.05275	

Pyrazines:5							
1	2,6-Dimethyl-3-ethylpyrazine	C_8_H_12_N_2_	136.2	1483.0	1224.884	1.22137	
2	2-Ethyl-5-methylpyrazine	C_7_H_10_N_2_	122.2	1434.7	1103.025	1.19929	
3	2-Methylpyrazine	C_5_H_6_N_2_	94.1	1277.6	785.716	1.10015	
4	2,3-Dimethylpyrazine	C_6_H_8_N_2_	108.1	1351.1	920.1	1.11023	
5	2,5-Dimethylpyrazine	C_6_H_8_N_2_	108.1	1326.8	872.824	1.11879	

Others:8							
1	3-Ethylpyridine	C_7_H_9_N	107.2	1387.6	995.983	1.1014	
2	*cis*-Rose oxide	C_10_H_18_O	154.3	1335.1	888.58	1.35394	
3	γ-Terpinene	C_10_H_16_	136.2	1260.9	758.995	1.21551	
4	2-Pentylfuran	C_9_H_14_O	138.2	1240.0	726.94	1.24647	
5	Thiophene	C_4_H_4_S	84.1	1032.9	397.105	1.03755	
6	Butylcyclohexane	C_10_H_20_	140.3	1035.2	399.8	1.26378	
7	Dimethyl sulfide	C_2_H_6_S	62.1	802.3	230.025	0.96027	
8	N,*N*-dimethylacetamide	C_4_H_9_NO	87.1	1412.7	1051.706	1.07089	

Note: RI: Retention index, Rt: Retention time, Dt: Migration time, [RIP rel] Normalized.

### 3.5 Dynamic principal component analysis (PCA) of GC-IMS

**Fig. 3:**
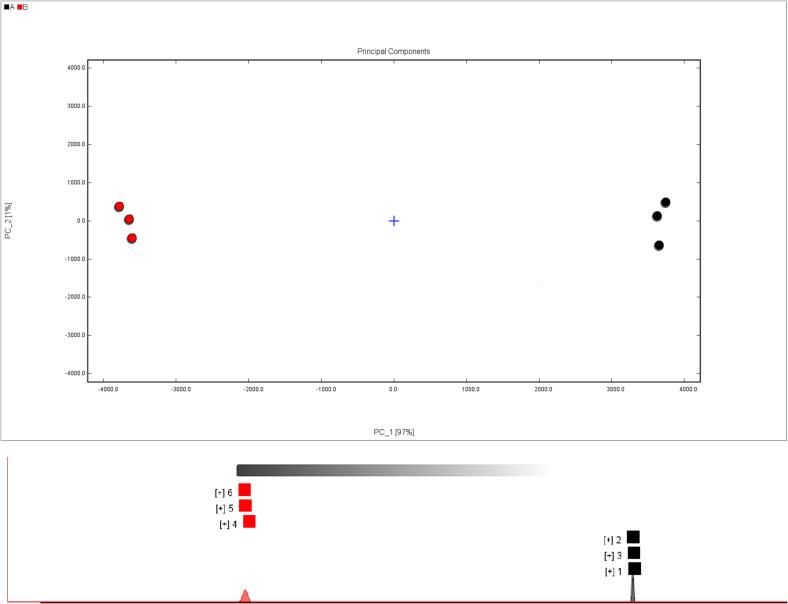
PCA analysis and “Nearest-neighbor” fingerprint analysis of GC-IMS (the closer the distance, the higher the similarity).

Dynamic PCA is based on the signal intensities of volatile compounds and can be used to distinguish between oil samples (Xu, Wang, et al., 2023). The PCA results are shown in Fig. 3, where the black and red dots (three replicates) represent the key volatile components of the purple and yellow passion fruit seed oils, respectively. In general, when the first two principal components are >60 %, the PCA model can separate the samples efficiently (Chen et al., 2020). The combination of principal component 1 (PC1, 97 %) and principal component 2 (PC2, 1 %) could effectively distinguish between the two different varieties of passion fruit seed oil. 98 % of the total variance was high, which indicates that PC1 and PC2 could explain almost all the flavor information. There was a large distance between the two oil samples along the PC1 axis, indicating that they are significantly different. This result is consistent with the sensory description of our oil extractors: purple passion fruit seed oil is associated with a fruity and floral attractive aroma, whereas yellow passion fruit seed oil has a rich oily and fat odor.

### 3.6 Qualitative analysis of volatile organic compounds (GC × IMS Library Search)

**Fig. 4:**
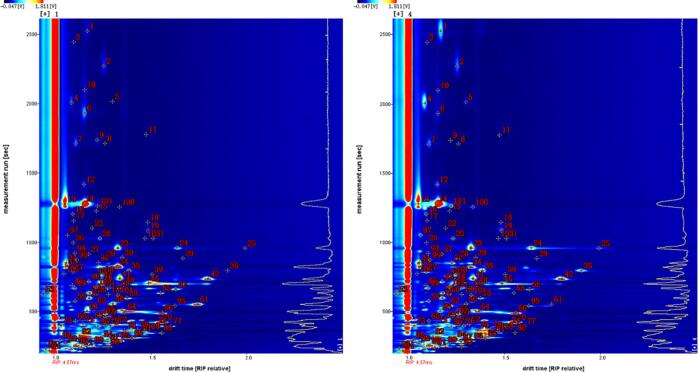
Qualitative analysis of volatile organic compounds via GC × IMS Library Search (A: purple passion fruit seed oil, B: yellow passion fruit seed oil). (For interpretation of the references to color in this figure legend, the reader is referred to the web version of this article.)

The volatile flavor substances in passion fruit seed oil were particularly enriched. A total of 108 volatile compounds were detected, including 23 alcohols, 21 esters, 17 aldehydes, 19 ketones, 9 alkenes, 6 acids, 5 pyrazines, and 8 others, such as hydrocarbons, ethers, furans, single sulfur compounds (dimethyl sulfide), and cycloalkanes (butylcyclohexane). In the case compounds exhibited two or three signal peaks, they were considered the monomers dimers or trimers. Generally, cold-pressed oil has a higher content of bioactive ingredients and stronger antioxidative characteristics, however, it lacks aroma due to inadequate Maillard reactions and delayed fatty acid oxidation in the absence of heating (Ramadan, 2013, Yin et al., 2021, Kozub et al., 2023). Except for the four most important aroma components (alcohols, esters, aldehydes, and ketones), the oil samples contained several specific odorous substances. Volatile compounds containing sulfur contribute to the unpleasant odors of cabbage, sulfur, asparagus, onion, putrid, and fish, which have been reported to be present in cold-pressed rapeseed oil (Liang et al., 2023) and roasted sesame oil (Yin et al., 2021) at high concentrations. Alkanes and acetoin reflect the odors of gasoline and butter, respectively, which are abundant in purple passion fruit seed oil (Liang et al., 2023). Thiophene, pinene, and pyridine are the main characteristic aromatic components of yellow passion fruit seed oil, giving garlicky, woody, and nutty aromas (Zhang, Chen, Zhang, et al., 2022, Xu et al., 2023). As shown in Fig. 4, each dot represents a compound, and the darker (red) the color, the more abundant the compound. In comparison, purple passion fruit seed oil contains particular odorous substances such as compound 34, acetoin (dimer), the simplest saturated ketone with a piquant odor; compound 64, 2-methyl-1-propanol (dimer), with a cortex odor; and compound 82, ethanol, with an alcoholic odor. The most characteristic flavor substances in yellow passion fruit seed oil are compound 1, acetophenone, containing both an aromatic and aliphatic group with a hawthorn odor, and compound 4, γ-Butyrolactone, with a 4-C closed ring and a creamy odor. These two flavor compounds (1 and 4) have been previously detected in walnut oil (Xu et al., 2023).

## 4 Conclusion

As a new type of plant oil resource, passion fruit seed oils exhibit potential for application in the food, medicine, and cosmetics industries. This study demonstrated the excellent physicochemical properties and beneficial fatty acid composition of cold-pressed passion fruit seed oils. High linoleic acid content is a key feature of passion fruit seed oil and is essential for maintaining the skin’s integrity, enhancing cell-membrane permeability, supporting the immune system, and assisting eicosanoid formation. A total of 108 volatile aroma compounds of passion fruit seed oils, mainly including four types of aldehydes, esters, alcohols, and ketones, were identified using HS-GC-IMS visual flavor fingerprinting technology. It’s amazing that abundant flavor there is in cold-pressed oils.

The composition and content of volatile compounds in seed oils from different varieties of passion fruits were found to be significantly different. The alcohol, ester, ketone, and acid contents, which contribute to flavors such as flowery, fruity, creamy, and yogurt, were higher in purple passion fruit seed oil. The contents of aldehydes, pyrazines, and alkenes related to fatty and nutty odors were higher in yellow passion fruit seed oil. PCA of GC-IMS revealed that such flavor compounds could be used to distinguish between two oils. Moreover, the results of GC × IMS Library Search showed that purple passion fruit seed oil contained acetoin (dimer) with a piquancy odor, 2-methyl-1-propanol (dimer) with a cortex odor, ethanol with an alcohol odor, acetophenone with a hawthorn odor, and γ-butyrolactone. Contrarily, yellow passion fruit seed oil was not rich in flavor substances, and the same was observed for the fruit flesh. The interpretation and evaluation of the flavor compounds of passion fruit seed oils can provide a theoretical basis for improving flavor quality and utilization. Nevertheless, volatile organic compounds have not been quantitatively and systematically analyzed using the proposed method; therefore, future work should include in-depth research using GC-GC TOF MS to fill this research gap. Our group is currently conducting such studies, and the findings will be reported in the future.

## CRediT authorship contribution statement

**Lili Zheng:** Formal analysis, Funding acquisition, Writing – original draft, Writing – review & editing. **Shenwan Wang:** Formal analysis, Investigation. **Yang Yang:** Resources. **Xiaoyan Zheng:** Investigation. **Dao Xiao:** Visualization. **Binling Ai:** Supervision. **Zhanwu Sheng:** Investigation, Supervision.

## Declaration of competing interest

The authors declare that they have no known competing financial interests or personal relationships that could have appeared to influence the work reported in this paper.

## Data Availability

Data will be made available on request.
